# Combined effects of weather conditions, transportation time and loading density on carcass damages and meat quality of market-weight pigs

**DOI:** 10.5194/aab-64-425-2021

**Published:** 2021-10-14

**Authors:** Nikola Čobanović, Saša Novaković, Igor Tomašević, Nedjeljko Karabasil

**Affiliations:** 1 University of Belgrade, Faculty of Veterinary Medicine, Department of Food Hygiene and Technology, Bulevar oslobodjenja 18, 11000 Belgrade, Serbia; 2 University of Belgrade, Faculty of Agriculture, Nemanjina 6, 11080 Belgrade, Serbia

## Abstract

This study investigated the interactive effects of weather conditions, transportation time and loading density on carcass damages and meat quality traits of market-weight pigs under commercial conditions. The following pork quality parameters were measured: pH and temperature; colour; drip, thawing and cooking loss; and textural traits. Pigs were assigned to one of eight groups arranged in a 2 × 2 × 2 factorial design according to the weather conditions (hot and cold), transportation time (short and long) and loading density (high and low). A three-way ANOVA and Tukey's post-test (multiple comparisons) were performed to assess the differences between groups in examined pork quality traits. Pigs exposed to short transportation (∼ 20 min) at high loading density (0.29 $m^{2}/100\;\mathrm{kg}$) during hot weather conditions produced meat with the lowest initial and ultimate meat pH value and sensory colour scores, and the highest initial temperature and the occurrence of pale, soft and exudative pork. The occurrence of pale, soft and exudative pork was reduced 5-fold during hot weather conditions when pigs were exposed to longer transportation (∼ 210 min) and low loading density (0.53 $m^{2}/100\;\mathrm{kg}$). Pigs exposed to short transportation (∼ 20 min) at high loading density (0.41 $m^{2}/100\;\mathrm{kg}$) during cold weather conditions produced the highest quality pork (the highest percentage of red, firm and non-exudative pork) characterised by lowest drip loss and $b^{*}$ value and the highest sensory colour scores. The highest percentages of carcass damages were recorded in pigs exposed to both low (0.50 $m^{2}/100\;\mathrm{kg}$) and high (0.33 $m^{2}/100\;\mathrm{kg}$) loading density during long transportation (∼ 210 min) in cold weather conditions. In conclusion, weather conditions and loading density are of greater importance for the occurrence of carcass damages and pork quality variation than transportation time.

## 1 Introduction

Consumer requirements for improved animal welfare in meat animals are having the most visible influence on their purchase behaviour worldwide (Faucitano et al., 2017; Tomašević et al., 2020). Pig transportation to the abattoir represents a critical control point in the pork production chain, because omissions and errors made at this point cause different degrees of stress and have negative repercussions on well-being, carcass and meat quality, which may neutralise all efforts made by the pig production sector to improve pig health, performance indices and welfare (Schwartzkopf-Genswein et al., 2012; Faucitano, 2018; Čobanović et al., 2020a). Earlier studies have demonstrated that transportation time, loading density and extreme weather conditions are the key aspects during transportation that can induce deleterious effects on health, welfare, carcass yield and ultimately pork quality (Scheeren et al., 2014; Čobanović et al., 2017; Rioja-Lang et al., 2019; Lemos Teixeira et al., 2020), but their results are inconsistent.

Some authors reported that synergistic effects between short transportation (< 1 h) and low loading density (0.50 m${}^{2}$/100 kg) cause acute antemortem stress and increase the incidence of pale, soft and exudative (PSE) pork characterised by abnormally low ultimate pH, light colour and reduced water-holding capacity (Guárdia et al., 2004). By contrast, other researchers found that pigs transported for a longer time (> 2 h) at smaller floor spaces (∼ 0.30 m${}^{2}$/100 kg) on the lorry may be more likely to develop PSE pork (Kim et al., 2004; Gajana et al., 2013). Several studies demonstrated that the interaction between long transportation time (> 2 h) and winter conditions contribute to the increased prevalence of carcass damages and higher occurrence of dark, firm and dry (DFD) pork with abnormally high ultimate pH, dark colour and increased water-holding capacity (Carr et al., 2008; Schwartzkopf-Genswein et al., 2012; Arduini et al., 2014; Scheeren et al., 2014). In contrast, the combination of high loading density and summer conditions has been shown to enhance antemortem glycogenolysis and lead to carcass damages, more developed *rigor mortis*, increased ultimate pH and darker colour, and thus tended toward DFD pork (Hunter et al., 1994). However, these results arise from studies where the effects of transportation time, loading density and weather conditions were assessed by combining the two factors. To the best of the authors' knowledge, the effects of all three factors together on carcass damages and meat quality traits have not yet been evaluated in a single study. Therefore, the aim of this study was to determine the interactive effects of weather conditions, transportation time and loading density on carcass damages and meat quality traits of market-weight pigs under commercial conditions.

## 2 Materials and methods

### 2.1 Animals, pre-slaughter conditions and slaughter procedure

The study was conducted in 2019 on 240 market-weight pigs (123 barrows and 117 gilts, average live weight of 107.60 ± 3.05 kg and 180 ± 8.5 d old) of the same genetics ([Yorkshire × Landrace] sows sired with Pietrain boars). The pigs were monitored through deliveries in eight consignments of 30 pigs (two consignments per season) in months representing traditional seasonal environments in the Republic of Serbia: January and February (winter), April and May (spring), July and August (summer), and October and November (autumn). Pigs originated from two commercial finishing farms with similar rearing conditions. On both farms, the pigs were raised in a finishing piggery on the fully slatted floor, in groups of 30 individuals, with a minimum of 1 m${}^{2}$/pig. Food and water were freely available 24 h a day at nipple drinkers and food dispensers during the finishing stage on both farms.

**Table 1:** The information about various pre-slaughter variables.

Batch	1	2	3	4	5	6	7	8
Number of pigs	30	30	30	30	30	30	30	30
Farm of origin	A	B	B	A	A	B	A	B
Season	Summer	Summer	Autumn	Autumn	Winter	Winter	Spring	Spring
Month	Jul	Aug	Oct	Nov	Jan	Feb	Apr	May
Average live weight per pig (kg)	103.86	113.33	108.89	105.26	109.09	106.47	108.84	105.15
Loading time (min)	53	62	68	50	45	63	55	70
Loading density ($m^{2}$/100 kg)	0.53	0.29	0.41	0.50	0.33	0.55	0.38	0.58
Transportation time (min)	225	22	20	210	210	25	230	20
Transport distance (km)	215	5	5	215	215	5	215	5
Waiting time (min)	10	15	11	5	8	20	10	5
Unloading time (min)	22	18	15	14	25	20	15	28
Lairage time (min)	60	65	63	61	62	65	68	60
Lairage density ($m^{2}$/pig)	0.65	0.65	0.65	0.65	0.65	0.65	0.65	0.65
Ambient temperature at unloading (${}^{\circ }C$)	28.0	34.5	9.9	0.4	7.3	5.2	21.5	22.2
Ambient relative humidity at unloading (%)	82.0	87.3	60.0	42.8	63.0	57.1	68.4	93.3
Lairage temperature (${}^{\circ }C$)	28.5	35.2	9.5	1.5	8.0	6.1	23.0	23.5
Lairage relative humidity (%)	85.5	88.8	55.8	44.5	66.5	60.00	70.00	92.0

Before transportation, pigs were not deprived of food or water. Pigs were loaded using a metal ramp (5 m length, slope ≤ 15${}^{\circ }$) on the same commercial single deck lorry by the same loading crew. Since the information was collected under commercial transport conditions, the loading density ranged from 0.29 to 0.58 $m^{2}/100\;\mathrm{kg}$, depending on the live weight and number of pigs in the lorry. Lorry departed from the farm immediately after loading. Transportation was carried out by the same driver and commercial single deck lorry. The pigs were directly transported from farm of origin to the abattoir, whereby the long transportation was always from farm A and short transportation from farm B. The lorry had neither drinking nipples nor showers, whereby side panels were 100 % open during transportation in all seasons (natural ventilation). The lorry floor was not bedded during transportation. The pigs were unloaded on arrival at the abattoir using a metal ramp (5 m length, slope ≤ 15${}^{\circ }$) and placed in a lairage pens for 1 h in groups of 30 individuals per pen (stocking density 0.65 m${}^{2}$/pig) with free access to water. Sprinklers and feeders were not available in lairage pens. Lairage temperature and relative humidity followed outside conditions. The driver was interviewed through the aid of structured questionnaires to collect information regarding farm of origin, loading time, transportation time, distance from farm to abattoir, loading density, average live weight per pig, waiting time and unloading time. In addition, the data about lairage time, lairage density and daily temperature and relative humidity (during unloading and lairage) were obtained from abattoir personnel. The information about various pre-slaughter variables is shown in Table 1. Slaughter of the pigs and processing of the carcasses were performed at the same commercial abattoir in compliance with the standard industry-accepted practices.

### 2.2 Sample size determination

The sample size calculation for each statistical test used was performed using the programme G*Power (version 3.1.9.7, University of Kiel, Germany) (Faul et al., 2007). For the power analysis using a F-test ANOVA (fixed effects, omnibus and one-way) with input parameters of effect size 0.45, α level 0.05, power 0.95 and eight groups, the required sample size (n) was a total of 120 pigs (15 pigs per group). For the power analysis using Z-test correlations (two independent Pearson r values) with input parameters of effect size 0.65, α level 0.05, power 0.95 and allocation ratio N2/N1 1, the required sample size (n) was a total of 110 pigs. For the power analysis using a $\chi^{2}$ goodness-of-fit tests (contingency tables) with input parameters of effect size 0.35, α level 0.05, power 0.95 and degrees of freedom (df) 7, the required sample size (n) was a total of 179 pigs.

### 2.3 Pork sampling procedure

All pork quality measurements were carried out 45 min, 24 and 72 h postmortem on the left carcass side at the position of the 10th and 11th ribs (*pars lumbalis*, central area of the loin) in the *musculus longissimus dorsi*. The abattoir was visited on two consecutive days once a month: on the first day, the initial meat pH (${\mathrm{pH}}_{i}$) and temperature ($T_{i}$) were measured in the cold chamber 45 min postmortem. On the following day, ultimate meat pH (${\mathrm{pH}}_{u}$) and temperature ($T_{u}$) were determined at the same site at 24 h postmortem. In addition, two boneless loins samples (each 2.54 cm thick, ∼ 100 g) were cut 24 h postmortem from each selected carcass by a trained abattoir personnel and used for subsequent determination of pork water-holding capacity and colour traits. At the same time interval and at the same anatomical location where meat samples were taken for water-holding capacity and colour measurements, a boneless loin sample (each 5 cm thick, ∼ 250 g) was cut from every second selected carcass (n = 120) for determination of pork textural traits. The samples to be used for determination of pork water-holding capacity, colour and textural traits were weighed on a semi-analytical scale, vacuum packed, kept on shaved ice in a cooler box and transferred to the laboratory for further analysis.

### 2.4 Determination of pork physicochemical traits

The initial and ultimate meat pH (${\mathrm{pH}}_{i}$ and ${\mathrm{pH}}_{u}$) and temperature ($T_{i}$ and $T_{u}$) of the *musculus longissimus dorsi* were measured using a pH meter (Testo 205, Testo AG, Lenzkirch, Germany). Meat pH and temperature were both determined in triplicate, and the average values of three measurements were taken as a final result.

### 2.5 Determination of pork water-holding capacity traits

Three methods (drip loss, thawing loss and cooking loss) were used for determination of pork water-holding capacity traits. For gravimetric estimation of drip loss based on the bag method (Honikel, 1998), each sample was weighed and suspended in a supporting mesh in a sealed polyethylene bag (with no contact between sample and polyethylene bag) and held at 4 ${}^{\circ }C$ for 48 h. After the storage period, samples were taken out of the polyethylene bag, exudate on the meat surface was carefully removed with tissue paper and weighed again. The drip loss was expressed as the percentage loss of the original weight of the boneless loins samples. Immediately after drip loss measurement, the same meat samples were suspended in a plastic freezer bag, frozen at -20 ${}^{\circ }C$ and stored for 48 h in a freezer. For analysis, all frozen meat samples were thawed at room temperature (20 ± 2 ${}^{\circ }C$) for 12–16 h. After thawing, exudate on the meat surface was gently wiped out with tissue paper and then the thawed meat samples were re-weighed. The thawing loss was estimated from the percentage weight ratio of before versus after thawing (Čobanović et al., 2020a). The thawed meat samples were separately packaged again using a plastic (Ziploc) bag, put in continuously boiling water bath and individually cooked to a central temperature of 75 ${}^{\circ }C$. During cooking, the internal temperature of meat samples was tracked with a thermometer with a hand probe (Testo 110, Testo AG, Lenzkirch, Germany). The cooked meat samples were removed from the water bath and cooled under chilled conditions (1–5 ${}^{\circ }C$) until equilibration. Thereafter, the cooled meat samples were taken from the bag, gently wiped out with tissue paper and then re-weighed. Differences of weights before and after cooking gave the cooking loss of the samples (Honikel, 1998).

### 2.6 Determination of pork colour traits

Instrumental and sensory pork colour measurements were both determined at 24 h postmortem after a standard 30 min blooming period. Instrumental colour was determined using a computer vision system (CVS) in triplicate according to Tomašević et al. (2019). CVS was calibrated as explained by Tomašević et al. (2021). Measurements were made by taking seven readings (technical replicates) on the surface and cross-section of each meat sample. The average $L^{*}$, $a^{*}$ and $b^{*}$ values of seven measurements were taken as a final result. The sensory colour of meat samples was evaluated by an analytical panel of three experienced sensorists based on the National Pork Producer Council (NPPC, 2000) colour standard, whereby colour scores ranged from 1 (pale colour) to 6 (dark colour).

### 2.7 Determination of pork textural traits

Pork textural traits in the form of texture profile analysis were performed using the TA.XT Plus Texture Analyzer (Stable Micro Systems Ltd., Surrey, UK) at room temperature with a 50 kg load cell. Data collection and calculations were made by using Exponent software (version 6.1.16.0, Stable Micro Systems, Surrey, UK). Before texture profile analysis, meat samples were grilled at 200 ${}^{\circ }C$ on a double-plate grill (Tefal OptiGrill+ GC3050, Rumilly, France) until the internal temperature reached 75 ${}^{\circ }C$. After grilling, meat samples were cooled at room temperature (20 ± 2 ${}^{\circ }C$) until equilibration. The textural profile analyses were performed using eight cylindrical cores from the middle (20 mm high and 12 mm diameter), positioned upright on a platform and compressed twice to 25 % of their original height (5 s delay between the first and second compression), in parallel to the muscle fibres, with a cylindrical aluminium probe (P/25). The pre-test speed and post-test speed were 180 mm/min, while the test speed was 60 mm/min,. The following textural traits were determined: hardness, springiness, cohesiveness and chewiness (Pons and Fiszman, 1996).

### 2.8 Determination of pork quality classes

Loins were classified into seven pork quality categories according to ${\mathrm{pH}}_{u}$ value, drip loss variation and $L^{*}$ value (Correa et al., 2007): (i) pale, soft and exudative (PSE) pork – ${\mathrm{pH}}_{u}$: < 5.5, drip loss (%): > 5, $L^{*}$ value: > 50; (ii) moderate pale, soft and exudative (moderate PSE) pork – ${\mathrm{pH}}_{u}$: 5.5–5.6, drip loss (%): > 5, $L^{*}$ value: ≥ 50; (iii) pale, firm and non-exudative (PFN) pork – ${\mathrm{pH}}_{u}$: 5.5–5.8, drip loss (%): < 5, $L^{*}$ value: > 50; (iv) red, soft and exudative (RSE) pork – ${\mathrm{pH}}_{u}$: 5.6–5.8, drip loss (%): > 5, $L^{*}$ value: 42–50; (v) red, firm and non-exudative (RFN) pork – ${\mathrm{pH}}_{u}$: 5.6–5.8, drip loss (%): 2–5, $L^{*}$ value: 42–50; (vi) moderate dark, firm and dry (moderate DFD) pork – ${\mathrm{pH}}_{u}$: 5.8–6.1, drip loss (%): < 5, $L^{*}$ value: 42–45; and (vii) dark, firm and dry (DFD) pork – ${\mathrm{pH}}_{u}$: > 6.1, drip loss (%): < 2, $L^{*}$ value: ≤ 42.

### 2.9 Determination of carcass damages

Carcass damages were visually assessed on the left carcass side by two trained observers in the cold chamber 45 min after slaughter using a visual scoring system based on Welfare Quality^®^ protocol (2009), as described in Čobanović et al. (2020a). Also, carcass damages were classified as handling-type carcass damages (large dark brown rectangular marks usually in the posterior part of the carcass typically caused by the sticks), fighting-type bruises (5–10 cm long, comma shaped and densely concentrated in the anterior and posterior parts of the carcass) and mounting-type carcass damages (10–15 cm long, 0.5 to 1 cm wide, densely concentrated on the back of pigs typically caused by the fore claws) by visual assessment of shape and size to recognise their origin (Faucitano, 2001).

### 2.10 Statistical analysis

Statistical analysis of the results was conducted with SPSS software (version 23.0, IBM Corporation, Armonk, NY, USA) (Nie et al., 2015). Before any formal statistical analysis, data were checked for linearity, normality of residuals (Shapiro–Wilk and Kolmogorov–Smirnov test), outliers and homogeneity of variance (Levene's test), and successfully passed all tests. Based on loading density, pigs were classified in two groups using the threshold for optimal loading density (0.425 m${}^{2}$/100 kg) recommended by EU regulations (European Commission, 2005): (i) high loading density: space allowance in the lorry lower than 0.425 m${}^{2}$/100 kg (n = 120); low loading density: the space allowance in the lorry higher than 0.425 m${}^{2}$/100 kg (n = 120), while animals were divided into two groups for transportation time: short transportation (< 30 min) (n = 120); and long transportation (> 210 min) (n = 120). Because ambient temperatures in each season were above or below the thermoneutral zone for market-weight pigs (10–21 ${}^{\circ }C$; Čobanović et al., 2020b), pigs were classified into two groups according to the season: (i) hot weather: ambient temperatures above the upper critical temperatures for market-weight pigs (pigs slaughtered in April, May, July and August) (n = 120); and (ii) cold weather: ambient temperatures below the lower critical temperatures for market-weight pigs (pigs slaughtered in November, December, January and February) (n = 120). Linear mixed model analysis was used with transportation time, loading density and season as fixed effects, while farm, gender and live weight were included in the model as random effects to correct for clustering within farms, genders and live weight. Data were analysed by fitting a linear mixed model using the restricted maximum likelihood method. Random effects were not significant (P > 0.05) and therefore were excluded from the final model. Multivariate analysis of variance (MANOVA) was performed to test the effects of weather conditions, transportation time and loading density and all possible interactions on different pork quality traits. Fixed effects and two-way interaction were not significant (P > 0.05) and therefore were excluded from the final model. Thus, three-way analysis of variance (ANOVA) at the 95 % confidence level was performed to test the combined effects of weather conditions, transportation time and loading density on different pork quality traits. Accordingly, pigs were assigned to one of eight groups arranged in a 2 × 2 × 2 factorial design according to the weather conditions (hot and cold), transportation time (short and long) and loading density (high and low). Significant means at P < 0.05 were further compared using Tukey's post-test (multiple comparisons). All results were described by descriptive statistics – mean value and pooled standard error of means (SEM). The $\chi^{2}$ test at the 95 % confidence level was additionally used to determine the percentage of pork quality classes and carcass damages with respect to the weather conditions, transportation time and loading density. Since none of the loins were classified as moderate DFD and DFD pork, these quality classes were not considered for this statistical test. Pearson correlations were calculated to determine the relationship between weather conditions, transportation time and loading density and carcass damages and pork quality traits. The Pearson correlation coefficient ($r_{p}$) was classified as weak (r|< 0.35), moderate (0.36 ≥|r|< 0.67) or strong (|r|≥ 0.68). Each pig was considered an experimental unit. A probability level of P < 0.05 was chosen as the limit for statistical significance in all tests.

**Table 2:** The combined effects of weather conditions, transportation time and loading density on pork quality traits (n = 240).

Weather conditions	Hot				Cold					
Transportation time	Short		Long		Short		Long			
Loading density	High	Low	High	Low	High	Low	High	Low	SEM	P value
Number of pigs	30	30	30	30	30	30	30	30		
Pork quality traits										
${\mathrm{pH}}_{i}$	5.83${}^{a}$	6.16${}^{b}$	6.08${}^{b}$	6.04${}^{b}$	6.07${}^{b}$	6.11${}^{b}$	6.23${}^{b}$	6.07${}^{b}$	0.13	< 0.0001
$T_{i}$ (${}^{\circ }C$)	38.92${}^{a}$	37.29${}^{b}$	36.50${}^{\text{bc}}$	37.92${}^{b}$	36.64${}^{\text{bc}}$	37.44${}^{b}$	37.10${}^{\text{bc}}$	37.66${}^{b}$	0.63	< 0.0001
${\mathrm{pH}}_{u}$	5.52${}^{a}$	5.83${}^{b}$	5.88${}^{b}$	5.71${}^{\text{bc}}$	5.83${}^{b}$	5.71${}^{\text{bc}}$	5.86${}^{b}$	5.80${}^{b}$	0.10	< 0.0001
$T_{u}$ (${}^{\circ }C$)	4.83	4.51	4.34	4.93	4.86	4.93	4.42	4.92	0.31	0.1861
Drip loss (%)	6.56${}^{a}$	5.19${}^{b}$	6.37${}^{a}$	5.26${}^{b}$	3.10${}^{c}$	5.27${}^{b}$	5.34${}^{b}$	4.53${}^{d}$	0.82	< 0.0001
Thawing loss (%)	6.20${}^{a}$	7.76${}^{b}$	6.14${}^{a}$	7.19${}^{b}$	4.62${}^{c}$	4.49${}^{c}$	4.81${}^{c}$	5.44${}^{c}$	0.80	0.0033
Cooking loss (%)	23.62${}^{a}$	23.58${}^{a}$	25.17${}^{a}$	24.45${}^{a}$	16.72${}^{b}$	23.84${}^{a}$	27.08${}^{a}$	15.62${}^{b}$	2.51	< 0.0001
$L^{*}$ value	58.33${}^{a}$	57.52${}^{a}$	52.51${}^{b}$	56.31${}^{a}$	51.76${}^{b}$	57.64${}^{a}$	53.23${}^{b}$	52.52${}^{b}$	2.75	0.0005
$a^{*}$ value	12.03${}^{a}$	17.75${}^{b}$	17.91${}^{b}$	15.12	16.54	14.52	15.50	15.23	3.61	0.0129
$b^{*}$ value	6.13${}^{a}$	6.56${}^{a}$	6.14${}^{a}$	5.62${}^{\text{ab}}$	4.35${}^{b}$	5.86${}^{\text{ab}}$	6.52${}^{a}$	5.50${}^{\text{ab}}$	0.83	0.0024
Sensory colour	1.15${}^{a}$	1.79${}^{b}$	1.71${}^{b}$	1.85${}^{b}$	2.41${}^{c}$	1.83${}^{b}$	1.69${}^{b}$	2.02${}^{b}$	0.25	< 0.0001
Hardness (N)	7.62${}^{a}$	1.94${}^{b}$	1.95${}^{b}$	3.53${}^{c}$	4.10${}^{c}$	4.09${}^{c}$	4.46${}^{c}$	3.72${}^{c}$	1.43	< 0.0001
Springiness (N)	0.70${}^{a}$	0.79${}^{b}$	0.76${}^{b}$	0.79${}^{b}$	0.77${}^{b}$	0.76${}^{b}$	0.76${}^{b}$	0.77${}^{b}$	0.03	< 0.0001
Cohesiveness (N)	0.70${}^{\text{ab}}$	0.63${}^{a}$	0.63${}^{a}$	0.70${}^{\text{ab}}$	0.71${}^{\text{ab}}$	0.78${}^{b}$	0.68${}^{a}$	0.66${}^{a}$	0.05	< 0.0001
Chewiness (N)	3.78${}^{a}$	0.96${}^{b}$	0.97${}^{b}$	1.96${}^{c}$	2.25${}^{c}$	2.30${}^{c}$	2.27${}^{c}$	1.86${}^{c}$	0.78	< 0.0001
Pork quality classes (%)										
PSE pork	66.67${}^{a}$	20.00${}^{b}$	20.00${}^{b}$	13.34${}^{\text{bc}}$	0.00${}^{\text{bc}}$	13.33${}^{\text{bc}}$	16.67${}^{\text{bc}}$	20.00${}^{b}$	–	< 0.0001
Moderate PSE pork	13.33${}^{a}$	50.00${}^{b}$	46.67${}^{b}$	23.33${}^{\text{ab}}$	13.33${}^{a}$	33.34${}^{\text{ab}}$	50.00${}^{b}$	16.67${}^{a}$	–	0.0005
RSE pork	6.67	3.33	16.66	0.00	0.00	10.00	6.67	16.66	–	0.0671
RFN pork	6.66${}^{a}$	6.67${}^{a}$	10.00${}^{a}$	13.33${}^{a}$	50.00${}^{b}$	10.00${}^{a}$	13.33${}^{a}$	16.67${}^{a}$	–	< 0.0001
PFN pork	6.67${}^{a}$	20.00${}^{a}$	6.67${}^{a}$	50.00${}^{b}$	36.67${}^{\text{ab}}$	33.33${}^{\text{ab}}$	13.33${}^{a}$	30.00${}^{\text{ab}}$	–	0.0003

Abbreviations: SEM – pooled standard error of means; ${\mathrm{pH}}_{i}$ – meat pH values measured 45 min postmortem; $T_{i}$ – meat temperature measured 45 min postmortem; ${\mathrm{pH}}_{u}$ – meat pH values measured 24 h postmortem; $T_{u}$ – meat temperature measured 24 h postmortem; drip loss – fluid loss at 4 ${}^{\circ }C$ for a period of 24 to 72 h (bag method); thawing loss – fluid loss during thawing at room temperature for 12–16 h; cooking loss – fluid loss during cooking until the internal temperature reached 75 ${}^{\circ }C$; ; $L^{*}$ value – light reflectance; $a^{*}$ value – intensity of red/green colour; $b^{*}$ value – intensity of yellow/blue colour; sensory colour – based on the NPPC (2000) colour standard (colour scores ranged from 1 (pale colour) to 6 (dark colour)); PSE – pale, soft and exudative; moderate PSE – moderate pale, soft and exudative; PFN – pale, firm and non-exudative; RSE – red, soft and exudative; RFN – red, firm and non-exudative. Note that significant differences were evaluated using the three–way ANOVA test and post hoc pairwise comparisons using Tukey's test. Different letters in the same row indicate a significant difference at P < 0.05 ${}^{\left(a-d\right)}$.

## 3 Results

### 3.1 The combined effects of weather conditions, transportation time and loading density on pork quality

Interactive effects of weather conditions, transportation time and loading density on pork quality traits are shown in Table 2. Three-way interaction (P < 0.0001) between weather conditions, transportation time and loading density affected pork physicochemical traits, whereby the lowest meat pH (${\mathrm{pH}}_{i}$ and ${\mathrm{pH}}_{u}$) and the highest meat temperature (Ti) were recorded in pigs subjected to high loading density (0.29 $m^{2}/100\;\mathrm{kg}$) during short transportation (∼ 20 min) in hot weather conditions.

Also, three-way interaction (P < 0.0001) between weather conditions, transportation time and loading density affected pork water-holding capacity traits, whereby the lowest drip loss was recorded in pigs subjected to high loading density (0.41 $m^{2}/100\;\mathrm{kg}$) during short transportation (∼ 20 min) in cold weather conditions (Table 2). Three-way interaction (P < 0.0001) between weather conditions, transportation time and loading density affected pork colour traits (Table 2). Pigs exposed to high loading density (0.29 $m^{2}/100\;\mathrm{kg}$) during short transportation (∼ 20 min) in hot weather conditions produced meat with the lowest sensory colour score. In contrast, pigs subjected to high loading density (0.41 $m^{2}/100\;\mathrm{kg}$) during short transportation (∼ 20 min) in cold weather conditions produced meat with the highest sensory colour score. In addition, three-way interaction (P < 0.0001) between weather conditions, transportation time and loading density affected pork textural traits (Table 2). Thus, the highest hardness and chewiness, but the lowest springiness were recorded in pigs subjected to high loading density (0.29 $m^{2}/100\;\mathrm{kg}$) during short transportation (∼ 20 min) in hot weather conditions. The combined effects (P < 0.0001) of weather conditions, transportation time and loading density showed that the highest occurrence of PSE pork was found in pigs subjected to high loading density (0.29 $m^{2}/100\;\mathrm{kg}$) during short transportation (∼ 20 min) in hot weather conditions (Table 2). On the contrary, pigs exposed to high loading density (0.41 $m^{2}/100\;\mathrm{kg}$) during short transportation (∼ 20 min) in cold weather conditions produced the highest (P < 0.0001) percentage of RFN pork (Table 2).

**Table 3:** The combined effects of weather conditions, transportation time and loading density on the prevalence of carcass damages (n = 240).

Weather conditions	Hot				Cold				
Transportation time	Short		Long		Short		Long		
Loading density	High	Low	High	Low	High	Low	High	Low	P value
Number of pigs	30	30	30	30	30	30	30	30	
Carcass damages (%)									
No carcass damages	36.67${}^{a}$	50.00${}^{a}$	46.67${}^{a}$	63.33${}^{a}$	56.67${}^{a}$	53.33${}^{a}$	3.33${}^{b}$	10.00${}^{b}$	< 0.0001
Moderate carcass damages	36.67	26.67	33.33	16.67	23.33	26.67	23.33	20.00	0.4787
Severe carcass damages	26.66${}^{a}$	23.33${}^{a}$	20.00${}^{a}$	20.00${}^{a}$	20.00${}^{a}$	20.00${}^{a}$	73.34${}^{b}$	70.00${}^{b}$	< 0.0001
Carcass damage type (%)									
Handling-type	13.33${}^{a}$	16.67${}^{a}$	16.67${}^{a}$	13.33${}^{a}$	13.33${}^{a}$	10.00${}^{a}$	3.33	63.33${}^{b}$	< 0.0001
Fighting-type	16.67${}^{a}$	13.33${}^{a}$	16.67${}^{a}$	6.67${}^{a}$	13.33${}^{a}$	10.00${}^{a}$	60.00${}^{b}$	13.33${}^{a}$	< 0.0001
Mounting-type	33.33	20.00	20.00	16.67	16.67	26.67	33.33	13.33	0.4116
Carcass regions (%)									
Ears	6.67	0.00	6.67	3.33	3.33	3.33	10.00	6.67	0.7552
Anterior part	16.67${}^{a}$	20.00${}^{a}$	13.33${}^{a}$	13.33${}^{a}$	16.67${}^{a}$	10.00${}^{a}$	63.33${}^{b}$	10.00${}^{a}$	< 0.0001
Middle part	20.00	10.00	20.00	6.67	10.00	16.67	16.67	10.00	0.6913
Posterior part	16.67${}^{a}$	13.33${}^{a}$	10.00${}^{a}$	13.33${}^{a}$	10.00${}^{a}$	13.33${}^{a}$	6.67${}^{a}$	56.67${}^{b}$	< 0.0001
Legs	3.33	6.67	3.33	0.00	3.33	3.33	0.00	6.67	0.7638

Note that significant differences were evaluated using the $\chi^{2}$ test. Different letters in the same row indicate a significant difference at P < 0.05${}^{\left(a-b\right)}$.

### 3.2 The combined effects of weather conditions, transportation time and loading density on the occurrence of carcass damages

Interactive effects of weather conditions, transportation time and loading density on the occurrence of carcass damages are shown in Table 3. The combined effects (P < 0.0001) of weather conditions, transportation time and loading density showed that the highest percentages of severe carcass damages were found in pigs subjected to both low (0.50 $m^{2}/100\;\mathrm{kg}$) and high (0.33 $m^{2}/100\;\mathrm{kg}$) loading density during long transportation (∼ 210 min) in cold weather conditions. Additionally, pigs subjected to high loading density (0.33 $m^{2}/100\;\mathrm{kg}$) during long transportation (∼ 210 min) in cold weather had the highest (P < 0.0001) percentage of fighting-type carcass damages and damages on the anterior part on the carcass. On the other hand, pigs that underwent low loading density (0.50 $m^{2}/100\;\mathrm{kg}$) during long transportation (∼ 210 min) in cold weather had the highest (P < 0.0001) percentage of handling-type damages and damages on the posterior part of the carcass.

**Table 4:** Pearson correlations between weather conditions, transportation time and loading density and carcass damages and pork quality traits.

Parameters	Ambient temperature			Ambient relative humidity			Transportation time			Loading density		
	(${}^{\circ }C$)			(%)			(min)			($m^{2}$/100 kg)		
	$r_{p}$	P value	Strength	$r_{p}$	P value	Strength	$r_{p}$	P value	Strength	$r_{p}$	P value	Strength
${\mathrm{pH}}_{i}$	-0.234${}^{*}$	< 0.0001	weak	-0.190${}^{*}$	0.004	weak	0.111	0.087	–	0.138${}^{*}$	0.0325	weak
$T_{i}$ (${}^{\circ }C$)	0.247${}^{*}$	< 0.0001	weak	0.189	0.003	weak	-0.102	0.113	–	-0.075	0.2468	–
${\mathrm{pH}}_{u}$	-0.268${}^{*}$	< 0.0001	weak	-0.187	0.004	weak	0.214${}^{*}$	0.0010	weak	0.097	0.1332	–
$T_{u}$ (${}^{\circ }C$)	-0.042	0.514	–	-0.089	0.171	–	-0.106	0.101	–	0.120	0.0632	–
Drip loss (%)	0.316${}^{*}$	< 0.0001	weak	0.249${}^{*}$	< 0.0001	weak	0.094	0.146	–	-0.197${}^{*}$	0.0022	weak
Thawing loss (%)	0.427${}^{*}$	< 0.0001	moderate	0.470${}^{*}$	< 0.0001	moderate	0.029	0.654	–	-0.177${}^{*}$	0.0061	weak
Cooking loss (%)	0.276${}^{*}$	< 0.0001	weak	0.331${}^{*}$	< 0.0001	weak	0.092	0.157	–	-0.144${}^{*}$	0.0253	weak
$L^{*}$ value	0.235${}^{*}$	< 0.0001	weak	0.287${}^{*}$	< 0.0001	weak	-0.231${}^{*}$	< 0.0001	weak	0.105	0.1057	–
$a^{*}$ value	-0.051	0.428	–	-0.007	0.917	–	0.052	0.423	–	0.090	0.1646	–
$b^{*}$ value	0.110	0.090	–	0.172	0.008	weak	0.065	0.317	–	-0.042	0.5174	
Sensory colour	-0.369${}^{*}$	< 0.0001	moderate	-0.362${}^{*}$	< 0.0001	moderate	0.021	0.749	–	0.233${}^{*}$	0.0003	weak
Hardness (N)	0.159${}^{*}$	0.050	weak	0.045	0.628	–	-0.204${}^{*}$	0.025	weak	-0.397${}^{*}$	< 0.0001	moderate
Springiness (N)	-0.045	0.627	–	0.099	0.283	–	0.027	0.771	–	0.352${}^{*}$	< 0.0001	weak
Cohesiveness (N)	-0.108	0.240	–	-0.130	0.159	–	-0.219${}^{*}$	0.016	weak	0.062	0.500	–
Chewiness (N)	0.157${}^{*}$	0.050	weak	0.020	0.827	–	-0.212${}^{*}$	0.020	weak	-0.344${}^{*}$	< 0.0001	weak
Carcass damages (%)	-0.253${}^{*}$	< 0.0001	weak	-0.240	< 0.0001	weak	0.247${}^{*}$	< 0.0001	weak	-0.167${}^{*}$	0.0097	weak

Abbreviations: ${\mathrm{pH}}_{i}$ – meat pH values measured 45 min postmortem; $T_{i}$ – meat temperature measured 45 min postmortem; ${\mathrm{pH}}_{u}$ – meat pH values measured 24 h postmortem; $T_{u}$ – meat temperature measured 24 h postmortem; drip loss – fluid loss at 4 ${}^{\circ }C$ for a period of 24 to 72 (bag method); thawing loss – fluid loss during thawing at room temperature for 12–16 h; cooking loss – fluid loss during cooking until the internal temperature reached 75 ${}^{\circ }C$; $L^{*}$ value – light reflectance; $a^{*}$ value – intensity of red/green colour; $b^{*}$ value – intensity of yellow/blue colour; sensory colour – based on the NPPC (2000) colour standard (colour scores ranged from 1 (pale colour) to 6 (dark colour)). Note that associations between ambient temperature, ambient relative humidity, transportation time and loading density and pork quality related parameters were determined using Pearson correlation. The Pearson correlation coefficient ($r_{p}$) was classified as weak (r|< 0.35), moderate (0.36 ≥|r|< 0.67) or strong (|r|≥ 0.68). Level of significance: ${}^{*}$ P < 0.05.

### 3.3 Pearson correlations between weather conditions, transportation time and loading density and carcass damages and pork quality traits

Pearson correlations between weather conditions, transportation time and loading density and carcass damages and pork quality traits are shown in Table 4. Ambient temperature and humidity weakly positively correlated (P < 0.0001) with initial meat temperature, drip loss, cooking loss and $L^{*}$ value. In addition, weak negative correlations were found between ambient temperature and humidity and initial (P < 0.0001) and ultimate meat pH (P = 0.004) and carcass damages (P < 0.0001). Ambient temperature and humidity moderately positively correlated (P < 0.0001) with thawing loss but moderately negatively correlated (P < 0.0001) with sensory colour scores. Transportation time weakly positively correlated with the ultimate meat pH value (P = 0.001) and carcass damages (P < 0.0001) but weakly negatively correlated with the hardness (P = 0.025), cohesiveness (P = 0.016) and chewiness (P = 0.020). Moderate negative correlations (P < 0.0001) were found between loading density and hardness and chewiness. In addition, loading density weakly negatively correlated with the drip loss (P = 0.0022), thawing loss (P = 0.0061), cooking loss (P = 0.0253), $L^{*}$ value (P = 0.028) and carcass damages (P = 0.0097) but weakly positively correlated with initial meat pH value (P = 0.0325) and sensory colour score (P = 0.0003).

## 4 Discussion

The present investigation revealed that pigs exposed to short transportation (∼ 20 min) at high loading density (0.29 $m^{2}/100\;\mathrm{kg}$) during hot weather conditions produced meat with the lowest initial and ultimate meat pH value, the lightest colour (the lowest sensory colour scores) and the highest occurrence of PSE meat (Table 2). As supporting evidence of these results, the increase in ambient temperature and humidity during unloading and lairage, as well as a decrease in available floor space in the lorry resulted in reduced initial and ultimate meat pH and sensory colour score but increased initial meat temperature, drip loss, thawing loss, cooking loss and $L^{*}$ value (Table 4). This can be explained by the fact that pigs did not have enough time and space to lie down and recover from the initial stressors induced by handling and loading procedures and to fully acclimatise to their new environment (Pilcher et al., 2011; Čobanović et al., 2016a; Faucitano and Goumon, 2018). In addition, ambient conditions on the day of slaughter were extremely hot and humid (34.5–35.2 ${}^{\circ }C$ and 87.3 %–88.8 %, Table 1), which made it even more difficult for the pigs to adapt to transport conditions, since they have limited ability to sweat due to dysfunctional keratinised sweat glands, thick subcutaneous fat, small heart and lungs (Čobanović et al., 2020b). Also, a high pig number and/or high mass of pigs per floor area during transportation may limit the air flow between them, reducing heat loss by convection and increasing air temperature inside the lorry that becomes higher than the external ambient temperature (Pereira et al., 2017). All these unfavourable transport conditions result in both acute stress and heat stress, which accelerate muscle metabolism and formation of lactic acid in the skeletal muscles. This leads to a very fast pH fall early postmortem, which together with high muscle temperature causes denaturation of sarcoplasmic and myofibrillar proteins and decreases water-holding capacity, subsequently resulting in the occurrence of PSE pork (Guàrdia et al., 2004; Hoffman and Fisher, 2010; Gajana et al., 2013; Gonzalez-Rivas et al., 2020). PSE pork is soft and moisture prior to heat treatment, while after cooking it lacks the juiciness, becomes harder and loses elasticity as a consequence of toughening of meat proteins due to their denaturation during abnormally fast muscle metabolism ante and postmortem (Barbut et al., 2005; Remignon et al., 2007; Öztürk and Serdaroglu, 2015; Dong et al., 2020). This can explain the toughest texture (the highest hardness and chewiness but lower springiness) being found in meat obtained from pigs subjected to short transportation (∼ 20 min) at low space allocation (0.29 $m^{2}/100\;\mathrm{kg}$) during hot weather conditions (Table 2). Based on the results obtained in this study, it can be argued that pigs transported over short distances at high loading density during hot weather conditions need more time in lairage to recuperate from transport-associated stress, which in turn would improve welfare and pork quality. It is suggested that 2–4 h is an optimal lairage time to reduce stress provoked during loading, transportation and unloading, and obtain better properties of pork (Čobanović et al., 2016b; Faucitano, 2018).

Compared to the pigs that underwent high loading density (0.29 $m^{2}/100\;\mathrm{kg}$) together with short transportation (∼ 20 min) in hot weather, those exposed to long transportation (∼ 210 min) at low loading density (0.53 $m^{2}/100\;\mathrm{kg}$) during the similar weather conditions produced meat with a higher initial (5.83 vs. 6.04) and ultimate meat pH (5.52 vs. 5,71), lower drip loss (6.56 % vs. 5.26 %), higher sensory colour score (1.15 vs. 1.85), tender texture (hardness: 3.53 vs. 7.62 N and chewiness: 1.96 vs. 3.78 N), which led to the significantly lower occurrence of PSE pork (66.67 % vs. 13.34 %) (Table 2). Most pigs begin to sit and lie down during the first half hour of transport, especially during hot weather conditions either due to heat exhaustion or in attempts to maximise heat loss through contact with the lorry floor or wall surface (Faucitano and Goumon, 2018; Rioja-Lang et al., 2019). Therefore, when provided more transportation time (> 3 h) with sufficient floor space, pigs may acclimate on transport conditions and partially recover from the stress induced during loading, so the transport vehicle could act as a resting place similar to the lairage pens in the abattoir, which is of great importance for decreasing the occurrence of PSE pork (Pérez et al., 2002; Chai et al., 2010; Faucitano and Goumon, 2018). Also, the results of this study confirmed that the recommended loading density (0.425 $m^{2}/100\;\mathrm{kg}$) during warmer weather conditions (> 25 ${}^{\circ }C$) should be decreased by 20 % to reduce transport stress and improve pork quality (Čobanović et al., 2016b; Nannoni et al. 2017).

In this study, pigs subjected to short transportation (∼ 20 min) at high loading density (0.41 $m^{2}/100\;\mathrm{kg}$) during cold weather conditions produced meat with better water-holding capacity (lower drip loss) and redder colour (lower $b^{*}$ value but higher sensory colour scores) compared with meat obtained from pigs exposed to other weather and transport conditions (Table 2). In addition, meat from half of the pigs from this group was classified as RFN pork (50.00 %), while more than one-third of the pigs produced PFN pork (36.67 %), and none of the pigs produced PSE pork (Table 2). The highest pork quality, obtained from before mentioned group of pigs, could be ascribed to the fact that the pigs were exposed to ambient temperature (9.5–9.9 ${}^{\circ }C$; Table 1) very close to the thermoneutral zone for market-weight pigs (10–21 ${}^{\circ }C$; Čobanović et al., 2020b). This indicates that pigs do not suffer from heat or cold stress and that they keep their body temperature constant without altering their normal behaviour and metabolic rate (Vermeer and Hopster, 2018). Moreover, those pigs were transported at the optimal floor space recommended by EU regulations (0.425 $m^{2}/100\;\mathrm{kg}$; European Commission, 2005), which allows each pig to stand or lie down in its natural position during transportation (Guàrdia et al., 2004). Therefore, pigs subjected to such weather and transport conditions probably experienced only mild stress, which caused a slowdown of metabolic processes in skeletal muscles postmortem, only partial denaturation of sarcoplasmic proteins and resulted in reduced occurrence of PSE pork and increased occurrence of PFN and RFN pork. Accordingly, the results of this study confirmed that when transport stress is mild, pork quality is not significantly affected (Sardi et al., 2020). It can be argued that transport quality in terms of selection of pigs fit for transportation, lorry design, loading density, weather conditions and road conditions is of much greater importance for animal welfare and meat quality than transportation time (Cockram, 2007).

The highest percentage of carcass damages was recorded in pigs exposed to low (0.50 $m^{2}/100\;\mathrm{kg}$) loading density during long transportation (∼ 210 min) in cold weather conditions (Table 3). In addition, pigs that underwent low loading density (0.50 $m^{2}/100\;\mathrm{kg}$) during long transportation (∼ 210 min) in cold weather had the highest frequencies of damages caused by inappropriate handling and damages on the posterior part of the carcass (Table 3). Over colder months, pigs spent more time standing during transportation to avoid the contact with the cold floor or wall surface, i.e. to reduce the surface area for heat loss (Goumon et al., 2013; Arduini et al., 2014; Scheeren et al., 2014). As a result of their standing posture during transportation, especially in combination with increased floor space in the lorry, pigs could not support one another, so they have difficulty in maintaining balance during accelerations, braking, turns of the lorry and on poor road surfaces (Scheeren et al., 2014). This indicates that providing pigs too much space during transportation does not necessarily result in more pigs lying down, but it causes more disturbance and aggression due to animals being able to move around, loss of balance and greater risk of being thrown around and getting stuck resulting in high frequency of carcass damages due to trampling and falls (Faucitano and Goumon, 2018; Rioja-Lang et al., 2019).

In the current study, the occurrence of carcass damages caused by fighting and damages on the anterior part on the carcass was the highest in pigs subjected to high loading density (0.33 $m^{2}/100\;\mathrm{kg}$) during long transportation (∼ 210 min) in cold weather conditions (Table 3). These results are supported because in this investigation the decrease in ambient temperature and space allowance in the lorry and increase in transportation time resulted in increased occurrence of carcass damages (Table 4). Aforementioned group of pigs was exposed to ambient temperatures (7.3–8.0 ${}^{\circ }C$; Table 1) a few degrees below the lower critical temperature for market-weight pigs (10 ${}^{\circ }C$; Čobanović et al., 2020b), which presumably triggered the mechanism of cold stress. Under conditions of cold stress, pigs exhibit huddling behaviour to create a warmer microclimate and to conserve body energy, which increases their ability to withstand low temperatures during transportation (Van de Perre et al., 2010a; Čobanović et al., 2016b). This huddling activity further reduces the available floor space in the lorry, which causes more fights or incidences of pigs climbing over the backs of other pen mates to find some room to lie down and rest, leading to higher frequencies of fighting-type carcass damages and damages on the anterior part on the carcass (Guàrdia et al., 2009; Van de Perre et al., 2010b; Pereira et al., 2017).

## 5 Conclusions

The results of this study showed that pigs exposed to short transportation (∼ 20 min) at low space allocation (0.29 $m^{2}/100\;\mathrm{kg}$) during hot weather conditions produced the lowest pork quality (the lowest initial and ultimate meat pH value and sensory colour scores but the highest occurrence of PSE pork). However, the occurrence of PSE pork was reduced 5-fold during hot weather conditions when pigs were exposed to longer transportation (∼ 210 min) and increased floor space (0.53 $m^{2}/100\;\mathrm{kg}$). On the other hand, pigs subjected to short transportation (∼ 20 min) at high loading density (0.41 $m^{2}/100\;\mathrm{kg}$) in cold weather conditions produced the highest quality pork (the highest percentage of RFN pork) in terms of colour and water-holding capacity. In addition, the highest percentages of carcass damages were recorded in pigs exposed to both low (0.50 $m^{2}/100\;\mathrm{kg}$) and high (0.33 $m^{2}/100\;\mathrm{kg}$) loading density during long transportation (∼ 210 min) in cold weather conditions. Based on the obtained results, it can be concluded that weather conditions and loading density are of greater importance for the occurrence of carcass damages and pork quality variation than transportation time. More research is needed to further investigate the effects of different transportation factors, such as weather conditions, genotype, pig weight, loading density and transportation time and their interaction, on pig behaviour, physiology and carcass and meat quality.

### Ethical statement

The study was performed under commercial conditions, whereby pigs were raised on two commercial finishing farms and slaughtered for human meat consumption at the commercial abattoir in compliance with the standard industry-accepted practices. Pigs were not exposed to any experimental invasive procedure *in vivo* (pork samples were taken from the carcasses, while carcass damages were assessed on the carcasses in the cold chamber). For these reasons, this experiment did not fall within the field of application of Directive 2010/63/EU on the protection of animals used for scientific purposes and therefore did not require a specific authorisation by the local animal welfare and ethical review body.

## Data Availability

The data that support the findings of this study are available from the corresponding author upon reasonable request.
